# Comparison of Habitat Selection Models Between Habitat Utilization Intensity and Presence–Absence Data: A Case Study of the Chinese Pangolin

**DOI:** 10.3390/biology14080976

**Published:** 2025-08-01

**Authors:** Hongliang Dou, Ruiqi Gao, Fei Wu, Haiyang Gao

**Affiliations:** Guangdong Provincial Key Laboratory of Silviculture, Protection and Utilization, Guangdong Academy of Forestry, Guangzhou 510520, China

**Keywords:** habitat characteristic, *Manis pentadactyla*, occurrence, habitat use, generalized additive model

## Abstract

Identifying habitat characteristics is essential for conserving critically endangered species. When quantifying species habitat characteristics, ignoring data types may lead to misunderstandings about species’ specific habitat requirements. This study focused on the critically endangered Chinese pangolin in Guangdong Province, China, and divided the study area into 600 m × 600 m grids based on its average home range. The burrow number within each grid was obtained through line transect surveys, with burrow numbers/line transect lengths used as direct indicators of habitat utilization intensity. The relationships with sixteen environmental variables, which could be divided into three categories, including topographic, human disturbance and land cover composition, were quantified using the GAM method. We also converted continuous data into binary data (0, 1), constructed GAMs and compared them with habitat utilization intensity models. Our results indicate that the habitat utilization intensity model identified profile curvature and slope as primary factors, showing a nonlinear response to profile curvature (*Edf* = 5.610, *p* = 0.014) and a positive relationship with slope (*Edf* = 1.000, *p* = 0.006). The presence–absence model emphasized distance to water (*Edf* = 1.000, *p* = 0.014), slope (*Edf* = 1.709, *p* = 0.043) and aspect (*Edf* = 2.000, *p* = 0.026). The intensity model explained significantly more deviance, captured complex nonlinear relationships and supported higher model complexity without overfitting. This study demonstrates that habitat utilization intensity data provides a more ecologically informative basis for in situ conservation (e.g., identifying core habitats), and the process from habitat selection to habitat utilization should be integrated to reveal species’ habitat characteristics.

## Simple Summary

This research compared how habitat utilization intensity data versus presence–absence data influences understanding the habitat preferences of the critically endangered Chinese pangolin (*Manis pentadactyla*) in Guangdong Province, China. Habitat utilization intensity data provides a more nuanced and ecologically informative picture of habitat characteristics than presence–absence data. Furthermore, the main environmental variables that drive habitat selection and utilization are different, and the process of habitat selection to utilization should be integrated to understand the specific habitat requirements. Habitat utilization intensity data better reveals how Chinese pangolins utilize habitats and the complex, nonlinear relationships with environmental factors. For precise conservation actions, habitat utilization intensity data should be prioritized where feasible.

## 1. Introduction

Defining habitat characteristics is essential for in situ conservation of critically endangered species [1,2]. Presence–absence data and habitat utilization intensity data might reflect specific responses to environmental variables and are both widely used to analyze species–habitat associations, assess habitat suitability, identify threat factors and guide targeted conservation practices [3,4]. However, the differences introduced by using different data types have often been overlooked. This can lead to inconsistencies in understanding the specific habitat requirements of endangered species, potentially hindering effective habitat conservation and management [5,6,7]. Essentially, presence–absence data indicates whether a species selects or avoids a particular habitat, while habitat utilization intensity quantifies the frequency and degree of habitat utilization, providing additional ecological details [8]. Therefore, comparing and integrating these two data types allows for a more comprehensive understanding of the ecological processes involved—from initial habitat selection to subsequent habitat utilization.

Currently, data on the intensity of habitat utilization by animal species are generally derived from the line transect method, the mark–recapture method and the relative abundance index (RAI) obtained by infrared camera traps [9,10,11]. However, the mark–recapture method is more applicable to small animal groups, such as rodents, but not applicable to highly protected and endangered species, which poses a risk of injury or death to animals. The RAI is calculated from infrared camera monitoring data and is more suitable for large- and medium-sized carnivores or herbivores that can be easily detected by infrared cameras. As animal activity traces cannot be accurately identified, the usual method of counting the frequency of individuals by the fixed length of line transects is only applicable during the daytime, and the efficiency of the field survey will be greatly undermined when the animals are sensitive to human activities.

The critically endangered Chinese pangolin is a typical example because it is a burrowing and solitary (except in the breeding period) animal species without leaving obvious footprints when active at night. Termites, such as *Odontotermes formosanus* and *Macrotermes barneyi,* are its important food resources [12]. Every burrow excavated by the Chinese pangolin was once a termite nest that was used for feeding or converted into a rest nest by the Chinese pangolin [13,14]. Therefore, a standardized count of burrows through the line transect method can serve as a direct quantitative indicator of habitat utilization intensity.

For presence–absence and habitat utilization intensity data, quantitative methods also differ [15]. Whereas in specific model construction, such as the Generalized Additive Model (GAM), the presence–absence data need to be transformed by a nonlinear link function, normally *Logit*, to constrain the range of predicted values (0, 1) and dynamically adjust the weights of the variables, on the other hand, habitat utilization intensity, which is defined as continuous data, can be directly modeled to address nonlinear relationships [16,17]. These two data types may respond differently to environmental variables, but there is a lack of case studies.

Here, we used the Chinese pangolin as an example to compare presence–absence and habitat utilization intensity data to quantify the differences in the habitat selection process based on the same set of environmental variables through establishing GAMs. Our study defined the differences and uncertainty of different data types in revealing the habitat selection characteristics and also provides a multidimensional and quantitative reference for the habitat selection and utilization mechanisms of the critically endangered Chinese pangolin.

## 2. Materials and Methods

### 2.1. Study Area and Field Investigation

**Figure 1:**
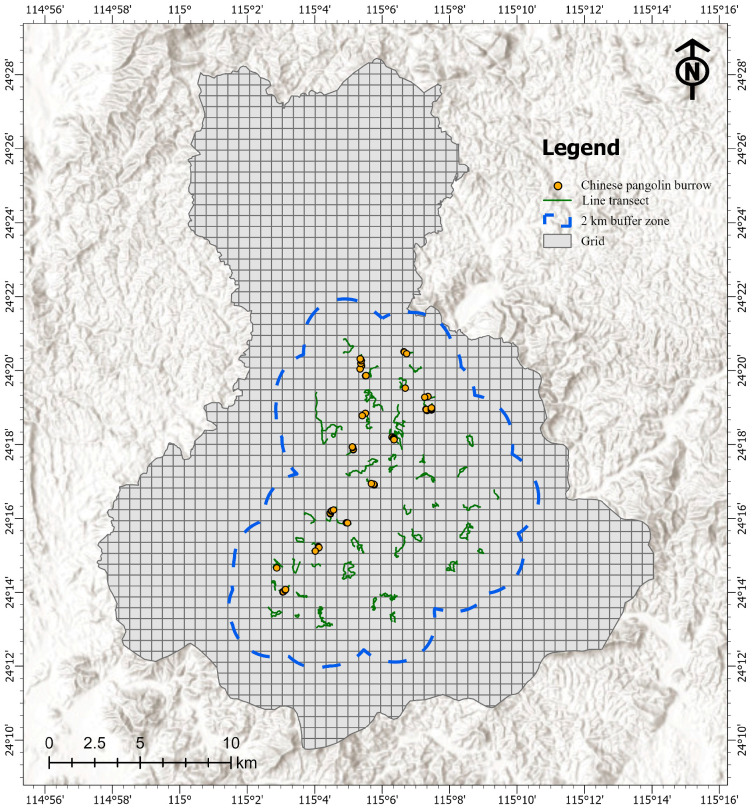
Distribution of line transects and Chinese pangolin burrows recorded in 600 m × 600 m grids in Heping County, Heyuan City, Guangdong Province, China.

Our study area involves four towns, including Dongshui, Lishi, Pengzhai and Linzhai, in Heping County, Heyuan City, China (Figure 1). This region is located in the typical subtropical monsoon climate zone, with a mild climate and an average annual temperature of 14–21 °C. Rainfall is abundant, with an average annual rainfall of >1000 mm. The landscape composition is mostly low-mountainous and hilly, and the forest vegetation covers about 75% of the total area. These forest areas are not ecologically protected, and there are a number of human settlements and cropland scatteredly distributed throughout the studied area.

We intermittently conducted field surveys of the Chinese pangolin populations from May 2022 to March 2023. All surveys were performed by a fixed, professionally trained field team, ensuring standardized data collection procedures and minimizing observer-related biases. We divided the study area into 600 m × 600 m grids with reference to the average home range of Chinese pangolins [17]. Based on the field topography, line transects of >600 m were set within 75 grids, reaching 32.60% of the total grids within the 2 km buffer zone, which is the core area where Chinese pangolins are distributed in this region, to record the locations and number of Chinese pangolin burrows observed. The number of burrows per unit length of line transect was used as a direct indicator of the habitat utilization intensity. We further transformed the intensity of the habitat utilization into binary data, assigning 1 to grids with burrow records and 0 to those without.

### 2.2. Environment Variable Acquisition and Screening

We downloaded DEM and land use data at a 30 m resolution from CAS Earth (https://data.casearth.cn/, accessed on 7 April 2025). We firstly calculated the land cover types and proportions within the 600 m × 600 m plots. Then, for continuous variables, we resampled the data to a 600 m resolution using the bilinear interpolation method to calculate the mean elevation, slope, aspect, profile curvature and plane curvature. There were a total of 16 environmental variables derived from those data that can be broadly categorized into three types, including topographic variables: mean elevation, slope, aspect, profile curvature and plane curvature; human disturbance variables: distance to human buildings, distance to cropland, percentage of human buildings and percentage of cropland; and land cover composition features: distance to water body, percentage of forest, percentage of shrubland, percentage of grassland, percentage of bare land, percentage of wetland and percentage of water body. Habitat utilization intensity, reassigned presence–absence data and environmental characteristics were tabled (Supplementary Table S1).

Highly correlated environmental variables (|*r*| ≥ 0.70) may cause statistical models to overfit [18]. Therefore, we firstly conducted a Spearman’s rank correlation analysis between 16 explanatory variables, and we further filtered those variables by two principles, retaining the maximum number of explanatory variables to preserve more data characteristics when the variables were highly correlated and selecting variables with more valid values (correlation analysis results: see Supplementary Table S2). Ultimately, 9 variables were screened out, including distance to water, distance to cropland, plane curvature, profile curvature, aspect, slope, altitude, percentage of shrubland and percentage of water body.

### 2.3. Model Fitting and Quantifying Variable Contribution Rate

We used the GAM method to separately construct models for these two types of dependent variable, since the responses of animals to environmental variables normally exhibit a certain degree of nonlinearity, such as ecological thresholds and tolerance ranges [19,20]. We introduced the ‘*offset*‘ term to the presence–absence model to eliminate the influence of the line transect length on the detection probability and avoid the bias of the model results. We set gradients of basis dimensions (*k*) until the model was overfitted. The *k* value is the number of basic functions used to capture the nonlinear relationships, and a large *k* value indicates that more cardinal units could be used to construct the smoothing function and that the model is more complex. Of particular note is that an excessively high *k* value may result in model overfitting. Conversely, a small *k* value probably fails to capture the complex structure and can lead to underfitting and low deviance explanation (DE) rates. We counted the values of *R*^2^, DE and AIC values at different *k* values. We also calculated the contribution rates of the variables to the models using ‘*glmm.hp*’ to compare the differences of the key influencing variables between the habitat utilization intensity and presence–absence models [21]. We plotted the response curves of significant variables. All analyses were performed in the *R* environment (version 4.3.2), and the ‘*mgcv*’ package (version 1.9-1) was mainly used to construct the GAMs [22].

## 3. Results

We cumulatively established 82.48 km of line transects within 75 sampled grids, with an average length of 1.10 km per grid, ranging from 0.61 km to 2.56 km. A total of 64 Chinese pangolin burrows were recorded within 16 grids, and no burrows were detected in the remaining 59 grids (Figure 1, Supplementary Table S1).

### 3.1. Differences Between Habitat Utilization Intensity and Presence–Absence Models

Habitat utilization intensity and presence–absence data show significant differences when fitted to the same set of environmental variables by the GAM method. In the habitat utilization intensity model, profile curvature (*Edf* = 5.610, *p* = 0.014) and slope (*Edf* = 1.000, *p* = 0.006) are significant variables and altitude is a marginally significant variable (*Edf* = 1.000, *p* = 0.071) (Table 1). However, in the presence–absence model, distance to water body (*Edf* = 1.000, *p* = 0.014), aspect (*Edf* = 2.000, *p* = 0.026) and slope (*Edf* = 1.709, *p* = 0.043) are significant variables (Table 1). The *R*^2^ values of the habitat utilization intensity and presence–absence models are 0.488 and 0.585, with deviance explained by 65.30% and 63.70%, respectively (Table 1).

**Figure 2:**
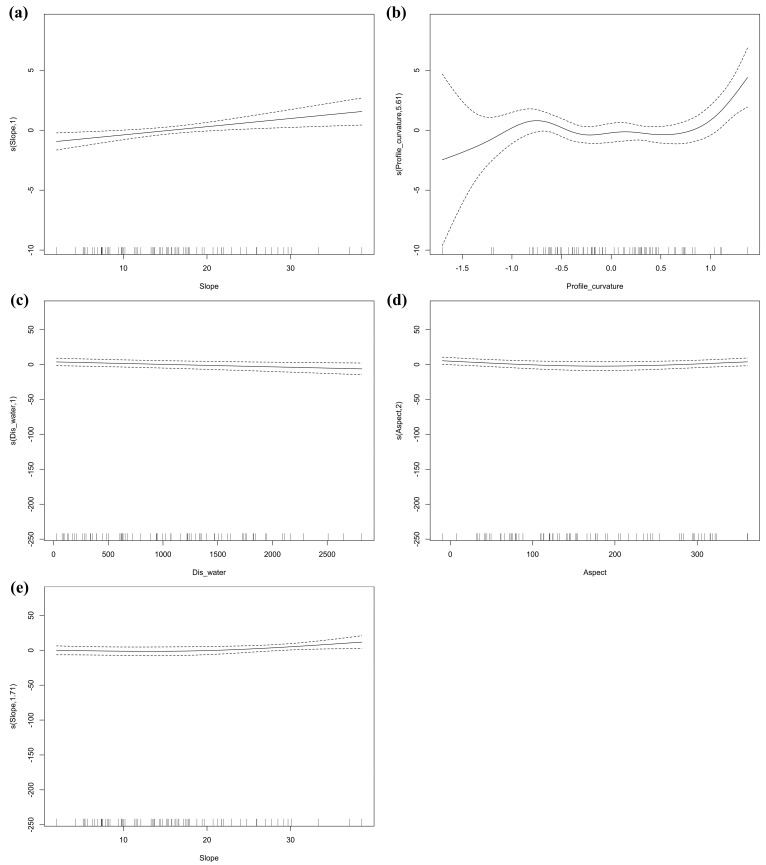
Response curves of significant variables to habitat utilization intensity: (**a**) slope and (**b**) profile curvature; response curves of significant variables to occurrence probability: (**c**) Dis_water (distance to water), (**d**) aspect, and (**e**) slope.

From the response curves, habitat utilization intensity increases with slope, as well as firstly exhibiting a nonlinear increasing trend with profile curvature, mainly concentrated in the −1 to 1 plateau (Figure 2a,b). The probability of Chinese pangolin occurrence shows a very slight decreasing trend with distance to water, slightly decreasing and then increasing with aspect, and a slightly increasing trend with slope (Figure 2c–e).

### 3.2. Response Differences of Environmental Variables

**Figure 3:**
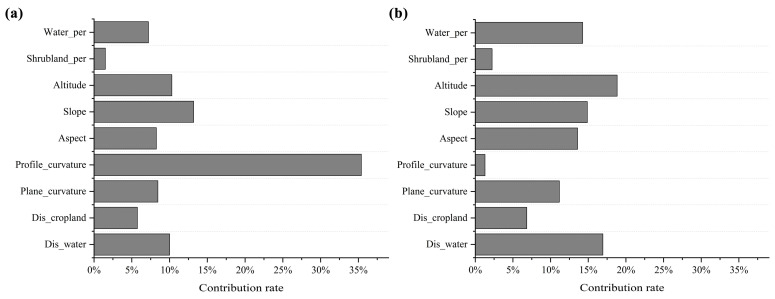
Contribution rates of primary environmental variables to the two models: (**a**) habitat utilization intensity model and (**b**) presence–absence model.

In the habitat utilization intensity model, the variable with the highest contribution rate is profile curvature (35.39%), followed by slope (13.18%), altitude (10.30%) and distance to water (10.01%) (Figure 3a). In the presence–absence model, the variable that contributes the most to the model is altitude (18.84%), followed by distance to water body (16.94%), slope (14.87%), percentage of water body (14.26%), aspect (13.58%) and plane curvature (11.17%) (Figure 3b). Other environmental variables contribute less than 10% to the models (Figure 3a,b).

### 3.3. Applicability of k Gradient and Its Effects on R^2^, DE and AIC

Our results show a more pronounced difference in the applicability of the two data types to *k* values, with a gradient of *k* values, ranging from 1 to 13, that can be applied to the habitat utilization intensity, with overfitting at *k* ≥ 14, and a gradient of *k* values, ranging from 1 to 3, that can be applied to the presence–absence data, with overfitting at *k* ≥ 4.

**Figure 4:**
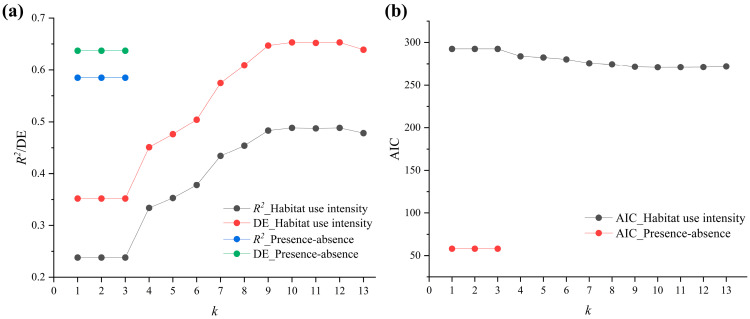
*R*^2^, DE and AIC variations under the set-of-*k* values gradient. (**a**) *R*^2^ and DE variations of habitat use intensity and presence–absence model, and (**b**) AIC variations of habitat use intensity and presence–absence model. *k* values range from 1 to 13 in habitat utilization intensity model and range from 1 to 3 in the presence–absence model.

Moreover, in the presence–absence model, whether *k* = 1, 2 or 3, its *R*^2^ (0.585), DE (63.70%) and AIC (56.86) values remain essentially unchanged (Figure 4a,b). The presence–absence model does not capture the apparent nonlinear relationship with related environmental variables, whereas in the habitat utilization intensity model, the values of *R*^2^ and DE increase with the increasing of *k*. At *k* = 10, *R*^2^ (0.488) and DE (65.30%) are the highest and the AIC value (271.01) is the lowest (Figure 4a,b).

## 4. Discussion

Quantifying the habitat characteristics of endangered species contributes to in situ conservation practices. In summary, our study reveals that different types of observation data can bring about differences in the understanding of habitat preferences, with the Chinese pangolin as an example, and these differences include the capture of nonlinear relationships, model complexity, the significance and contribution of the related environmental variables and the explanatory rate of the models.

Analyzing the presence–absence and habitat utilization intensity data reveal habitat characteristics throughout the ecological processes from habitat selection to utilization. In this study, the Chinese pangolin might prefer grids with available water sources and specific slope positions, concentrated between profile curvatures of −1 and 1, with habitat utilization intensity gradually increasing as the slope increases. The primary reason for this is possibly that moderate curvature (−1 to 1) ensures both burrow site safety and foraging efficiency while moderate slope and proximity to water sources further optimize available resources [23]. A moderate slope also helps avoid predators and human disturbance [24,25,26]. These factors interact to collectively form the core habitat utilization pattern of the Chinese pangolin in this region.

The two data types reveal the habitat characteristics of the Chinese pangolin from different perspectives. Compared with binary data (presence–absence data), continuous data (habitat utilization intensity) has advantages in capturing nonlinear relationships, particularly in terms of information density and expressive flexibility, for continuous variables contain ordered numerical information, with each value providing a unique data point, whereas binary data lose the details of those variations both within and outside the threshold [27,28]. Secondly, continuous data allow for smoother and more complex functional forms, whereas binary data normally produce a discrete ‘jump volatility’ effect in the model-building process, lacking in model complexity and flexibility [29]. Therefore, in this study, the habitat utilization intensity model can capture the nonlinear relationship with profile curvature while the presence–absence model cannot.

The two data types have their own advantages and disadvantages when used in fitting GAMs. The pseudo-*R*^2^ is relatively high in the presence–absence model; this may be due to differences in the calculation method. The pseudo-*R*^2^ of the binary model is based on the likelihood ratio calculation, and its value is significantly influenced by data distribution and model structure [30]. When the model can effectively distinguish between presence and absence, the pseudo-*R*^2^ may be relatively high (e.g., 0.585 in this example), but its actual predictive capability may not be superior to that of the continuous model. The deviance explanation rate reflects the proportion of uncertainty in the response variable reduced by the model and can be directly compared across GAMs [31]. The continuous model explains 65.30% of the deviance despite a lower *R*^2^. The binary model explains 63.70% deviance, and despite its higher pseudo-*R*^2^, its overall explanatory power is slightly weaker.

Therefore, when aiming to capture the complex nonlinear relationships between independent and dependent variables, retaining the continuous form of variables is typically a preferred strategy. Continuous data provides the information density and mathematical flexibility required for the model to define the true patterns of the data [8,10]. While binary data simplifies the problem, it comes at the cost of losing a significant amount of information and limiting the model’s expressive power, often resulting in a substantial decline in the ability to capture nonlinear relationships and potentially introducing bias due to artificially set thresholds [10]. Therefore, in habitat analysis, habitat utilization intensity data is preferred to capture richer ecological gradient information; a binary data based model (presence/absence) can be used as a supplement, suitable for large-scale distribution prediction or data-limited scenarios.

A limitation of this study is that burrows were not fully counted, potentially underestimating the intensity of habitat utilization by species, but the quantitative results of relative utilization intensity remain reliable. Ultimately, our results suggest that habitat utilization intensity data can support a larger number of cardinal dimensions than presence–absence data, constructing more complex models and capturing nonlinear relationships.

## 5. Conclusions

Our study demonstrates that data type profoundly influences the interpretation of habitat characteristics for the critically endangered Chinese pangolin. Habitat utilization intensity (continuous) data outperformed presence–absence (binary) data in detecting nonlinear relationships, achieving higher deviance explained despite a lower *R*^2^. Continuous data supported a more complex model structure without overfitting, revealing key ecological gradients. Critically, significantly environmental variables vary between the models, and these differences highlight that continuous data captures more nuanced habitat utilization patterns than binary data, linking habitat selection to utilization intensity. For precise in situ conservation, such as identifying core habitats or mitigating human disturbance in a specific small region, habitat utilization intensity data should be prioritized where feasible. Presence–absence data also remains useful for broad-scale distribution mapping but obscures utilization mechanics. Future studies on burrowing species, like the Chinese pangolin, should adopt continuous indicators to unlock deeper ecological insights.

## Figures and Tables

**Table 1:** Comparisons of the habitat utilization intensity model and presence–absence model. *, *p* < 0.05; **, *p* < 0.01. Dis_water: distance to water, Dis_cropland: distance to cropland, Shrubland: percentage of shrubland, Water: percentage of water body.

	*Edf*	*Ref.df*	*F*	*p-*Value	*R* ^2^	Deviance Explained
Habitat utilization intensity model					0.488	65.30%
s(Dis_water)	1.000	1.000	2.785	0.101		
s(Dis_cropland)	6.378	7.331	1.323	0.268		
s(Plane_curvature)	2.749	3.412	1.366	0.197		
s(Profile_curvature)	5.610	6.582	2.802	0.014 *		
s(Aspect)	2.136	2.657	1.808	0.193		
s(Slope)	1.000	1.000	8.258	0.006 **		
s(Altitude)	1.000	1.000	3.399	0.071		
s(Shrubland)	1.000	1.000	0.076	0.784		
s(Water)	2.959	3.565	1.860	0.107		
Presence–absence model					0.585	63.70%
s(Dis_water)	1.000	1.000	6.077	0.014 *		
s(Dis_cropland)	1.000	1.000	0.232	0.630		
s(Plane_curvature)	1.858	1.977	4.381	0.138		
s(Profile_curvature)	1.000	1.000	0.326	0.568		
s(Aspect)	2.000	2.000	7.306	0.026 *		
s(Slope)	1.709	1.909	5.377	0.043 *		
s(Altitude)	2.000	2.000	4.394	0.111		
s(Shrubland)	1.000	1.000	1.758	0.185		
s(Water)	1.666	1.885	1.874	0.440		

## Data Availability

The raw data used in this study are all listed in the Supplementary Materials (Table S1).

## Supplementary Materials

The following supporting information can be downloaded at: https://www.mdpi.com/article/10.3390/biology14080976/s1, Table S1: Raw data used to conduct correlation analysis and model construction; Table S2: Correlation analysis of 16 environmental variables.
